# Clinical implementation of multisequence MRI‐based adaptive intracavitary brachytherapy for cervix cancer

**DOI:** 10.1120/jacmp.v17i1.5736

**Published:** 2016-01-08

**Authors:** Jacqueline E. Zoberi, Jose Garcia‐Ramirez, Yanle Hu, Baozhou Sun, Carol G. Bertelsman, Pawel Dyk, Julie K. Schwarz, Perry W. Grigsby

**Affiliations:** ^1^ Department of Radiation Oncology Washington University School of Medicine St. Louis MO USA

**Keywords:** magnetic resonance imaging, brachytherapy, cervix cancer, high dose rate, adaptive

## Abstract

The purpose of this study was to describe the clinical implementation of a magnetic resonance image (MRI)‐based approach for adaptive intracavitary brachytherapy (ICBT) of cervix cancer patients. Patients were implanted with titanium tandem and colpostats. MR imaging was performed on a 1.5‐T Philips scanner using T2‐weighted (T2W), proton‐density weighted (PDW), and diffusion‐weighted (DW) imaging sequences. Apparent diffusion coefficient (ADC) maps were generated from the DW images. All images were fused. T2W images were used for the definition of organs at risk (OARs) and dose points. ADC maps in conjunction with T2W images were used for target delineation. PDW images were used for applicator definition. Forward treatment planning was performed using standard source distribution rules normalized to Point A. Point doses and dose‐volume parameters for the tumor and OARs were exported to an automated dose‐tracking application. Brachytherapy doses were adapted for tumor shrinkage and OAR variations during the course of therapy. The MRI‐based ICBT approach described here has been clinically implemented and is carried out for each brachytherapy fraction. Total procedure time from patient preparation to delivery of treatment is typically 2 hrs. Implementation of our technique for structure delineation, applicator definition, dose tracking, and adaptation is demonstrated using treated patient examples. Based on published recommendations and our clinical experience in the radiation treatment of cervix cancer patients, we have refined our standard approach to ICBT by 1) incorporating a multisequence MRI technique for improved visualization of the target, OARs, and applicator, and by 2) implementing dose adaptation by use of automated dose tracking tools.

PACS numbers: 87.61.‐c, 87.53.Jw, 87.19.xj

## I. INTRODUCTION

Three‐dimensional (3D)‐based intracavitary brachytherapy (ICBT) treatment planning for cervical cancer was implemented by the Groupe Européen de Curiethérapie‐European Society for Radiotherapy & Oncology (GEC‐ESTRO) working group, providing guidelines on the use of magnetic resonance imaging (MRI), primarily T2‐weighted (T2W) sequences, for target definition.^1^, ^2^ These guidelines have been implemented at some institutions with access to MRI units and, over time, have facilitated a transition from Point A‐based dose prescriptions to dose‐volume adaptation.^3^, ^4^, ^5^, ^6^, ^7^ An increase in local control and improvement in overall survival using MRI‐assisted, dose‐volume adaptation for ICBT, combined with 3D conformal EBRT, has been reported by one of these institutions.^8^, ^9^

At our institution, our standard approach for cervix cancer treatment — 25–28 fractions of positron emission tomography/computed tomography intensity‐modulated radiation therapy (PET/CT‐guided IMRT) combined with six weekly fractions of either film‐based or CT‐based high dose rate (HDR) ICBT using standard source distribution rules normalized to Point A—was performed from 1997–2008 and the results of this approach have been published previously.^(10)^ Since then, we have found that T2W‐MRI fused with other MRI sequences can assist with target volume delineation (via diffusion‐weighted imaging, DWI) and with applicator reconstruction (via proton density‐weighted imaging, PDW).^11^, ^12^, ^13^ We have used this multisequence MRI technique for ICBT treatment planning in addition to FDG‐PET/CT imaging for IMRT treatment planning, and have reported our analysis of dose‐volume parameters predicting gross tumor volume (GTV) control using this treatment technique.^(14)^ Total dose delivered to the GTV from combined MRI‐based HDR and PET/CT‐guided IMRT was found to be highly correlated with local tumor control.^(14)^ With this information in hand, we have further refined our brachytherapy treatment technique to include dose tracking and adaptation, similar to what has been done by institutions that have implemented GEC‐ESTRO recommendations.^8^, ^15^

Here we describe the implementation of the multisequence MRI technique using T2W, DW, and PDW sequences for ICBT planning of cervix cancer patients. We also describe how we perform brachytherapy dose adaptation by tracking tumor volumes, tumor doses, and organs at risk (OAR) doses over the course of treatment, using an automated dose‐tracking application.^(16)^ We have clinically implemented these methods at our facility and, using treated patients as examples, we demonstrate features of our MRI‐based technique for adaptive ICBT.

## II. MATERIALS AND METHODS

### A. Treatment prescription guidelines

All cervix cancer patients underwent FDG‐PET/CT simulation and were treated with IMRT, as described previously.^(10)^ Contoured nodal areas included the pelvic, inguinal, and if involved, para‐aortic lymph nodes with a 0.7 cm expansion excluding bony anatomy to form a nodal clinical target volume (CTVnodal). Metabolically active lymph nodes were contoured as a nodal metabolic target volume (MTVnodal). The nodal planning target volume (PTVnodal) included the CTVnodal expanded by 0.5 cm, and then added to the MTVnodal. The MTVcervix was defined as the 40% threshold volume.^(17)^ Patients were prescribed to receive IMRT treatment to the MTVcervix, MTVnodal, and PTVnodal to the dose levels specified in Table 1 in 1.8 Gy fractions four days per week with HDR brachytherapy delivered one day of the week, in six weekly fractions. Below, we describe in more detail the techniques implemented for each brachytherapy fraction.

**Table 1:** Treatment guidelines for carcinoma of the intact cervix.

		*IMRT* ^a^			
					*HDR Brachytherapy*
*Tumor Stage*		*MTV Cervix (cGy)*	*PTV Pelvic Nodes (cGy)*	*Para‐Aortic and/or Pelvic MTV Nodal (cGy)*	*Dose to Point A (cGy) in 6 Fractions*
Carcinoma *in situ*	*in situ* Ia1	0	0	0	3420
Ia2, Ib	<10 cc	0	4500	0	3420
Ib1, IIa	(10–30 cc)	1000	5040	5040	3420
Ib2, IIa, IIb	(30–60 cc)	2000	5040	5040	3900
IIb, III	(60–120 cc)	2000	5040	5040	4140
III	>120 cc	2000	5040	5040	4380
IIB, IIIB, IV	Suboptimal Brachytherapy due to tumor anatomy	7020	4992	4992	0

^a^ PTV fractionation is 180 cGy per fraction with MTV Cervix treated simultaneously.

### B. Imaging

All patients reviewed an MRI safety questionnaire prior to each implant. Patients were implanted with a titanium tandem and colpostat applicator (Varian Medical Systems, Inc., Palo Alto, CA) in a semisterile environment. Either saline‐soaked gauze or saline‐filled balloon packing (Alatus Vaginal Balloon Packing System, Radiadyne LLC., Houston, TX), was used to immobilize the implanted applicator within the patient, as well as provide distance between the applicator and adjacent OARs. Prior to imaging, 1 mg glucagon subcutaneous was also given to some patients to reduce bowel motion. Imaging was performed on a 1.5‐T MR scanner (Intera, Philips Medical Systems, Inc., Cleveland, OH). Patients were positioned supine with body/pelvis phased array surface coils. MR scanning sequences included T2W turbo spin echo (TSE) (TR=(3200−6500)ms,TE=100 ms, in‐plane resolution 0.1 cm, section thickness 0.5 cm, sagittal and axial acquisition planes), single‐shot DW echoplanar (TR=1300 ms,TE=75 ms, in‐plane resolution 0.2 cm, section thickness 0.5 cm, sagittal acquisition planes) at b values of 0 and 800 s/mm^2^, with diffusion gradients applied in all three directions, and PDW TSE (TR=(3000−6000) ms,TE=5.5 ms, in‐plane resolution 0.1 cm, section thickness 0.25 cm, sagittal acquisition planes), zero gap, foldover in the foot–head direction, 3–6 min per sequence. Imaging extents were set approximately from L4 to the ischial tuberosities in the craniocaudal direction, from the abdominal wall to the sacrum in the anterior–posterior direction, and were set to include the iliac crests in the transverse direction. Apparent diffusion coefficient (ADC) maps were generated from the DW‐MRI on the scanner console software in the following manner:

(1) $$ADC\;value=\frac{1}{b}\text{ln}\left(\frac{So}{S_{DW}}\right)$$

where $S_{DW}$ and *So* are signal intensities measured with and without diffusion‐weighted gradients, respectively, and the *b* value represents the diffusion factor.^(18)^ It has been shown previously that ADC values in tumors are generally less than those in benign tissues, appearing hypointense in the images.^18^, ^19^, ^20^, ^21^

### C. Treatment planning

T2W images, PDW images, and ADC maps were transferred to a radiation therapy treatment planning system (BrachyVision TPS, Varian Medical Systems, Inc., Palo Alto, CA) for contouring and isodose planning. Because the images were acquired in the same imaging session, the datasets upon import into the TPS were automatically registered based on their shared frame of reference and then manually verified using the applicator and surrounding anatomy. T2W images were utilized for contouring of OARs (i.e., bladder, rectum, and sigmoid). Point A was defined relative to the applicator using the classical definition on the T2W images.^(7)^ The delineation of the GTV, defined as the primary cervical gross tumor volume, was generally carried out on the T2W images, then verified against the registered ADC map and, if necessary, adjusted to improve agreement. The PDW images were used for reconstruction of the intrauterine tandem and the vaginal colpostats using the signal void resulting from the titanium applicator.^(22)^ Forward treatment planning was performed using standard source distribution rules with a dose of 6.5 Gy/fraction normalized to Point A.

### D. Dose tracking and adaptation

The Digital Imaging and Communications in Medicine (DICOM) standard radiation therapy (RT) dose, plan, and structure files were exported from the TPS to an automated dose tracking application referred to as the “HDR Dose Tool” (MATLAB, MathWorks, Inc., Natick, MA). The HDR Dose Tool was used to create an HTML‐based spreadsheet which tracked external beam dose and brachytherapy dose, as well as cumulative doses.^(16)^ Some examples of dose tracking parameters recorded on the spreadsheet included the mean Point A dose, target volume dose metrics (e.g., mean dose, volume of GTV receiving prescription dose (V100), and doses to 100% and 90% of GTV (D100 and D90)), and the minimum doses to the maximally exposed 2 cm^3^ of the OARs (D2cc). The doses were also normalized to their equivalent dose in 2 Gy fractions (EQD_2_).^(15)^

Dose adaptation may have been performed if the fractional D2cc to the OARs was greater than some threshold (i.e., 70%–80% of mean Point A dose) and if the cumulative D2cc was greater than some threshold ($90\;{\text{Gy}}_{\alpha \beta 3}$ for bladder, $75\;{\text{Gy}}_{\alpha \beta 3}$ for rectum and sigmoid, as recommended by other adopters of MR‐based ICBT).^8^, ^15^ Dose adaptation, if necessary, was carried out via two different approaches. The first, more traditional, approach involved making modifications in applicator geometry (e.g., mini‐ovoids used in place of standard‐sized ovoids to reduce OAR doses). This form of dose adaptation, referred to as “applicator optimization”, could be performed with or without “weighting optimization” where all dwell weights resulting from the standard source distribution rules were scaled down uniformly.^(8)^ Weighting optimization was usually performed after fraction 3, given adequate tumor volume shrinkage (> 50% reduction), and usually involved decreases in dwell weights of either 10% or 20%, while maintaining the V100 for that fraction ≥90%. Thus, weighting optimization yielded symmetric decreases of the dwell times with the dwell positions left intact. Even if the doses to OARs were greater than the respective thresholds in the first half of treatment, tumor dosage took priority, and the standard source distribution rules were followed for the first half of treatment.

## III. RESULTS

Below we demonstrate the use of our techniques on three of our patients who were enrolled on a research protocol approved by the Human Research Protection Office.

### A. Examples for the delineation of OARs and GTV

**Figure 1:**
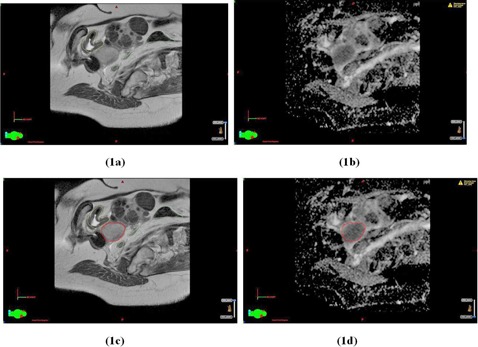
A parasagittal slice in the T2W image dataset (a) and the corresponding slice in the ADC map (b) about 1.5 cm lateral to the tandem for Patient 1. The same slices in the T2W image dataset (c) and ADC map (d) displayed with the contour for GTV (in red). Organ‐at‐risk contours for bladder (yellow) and sigmoid (light green) are displayed on the T2W images (a) and (c).

**Figure 2:**
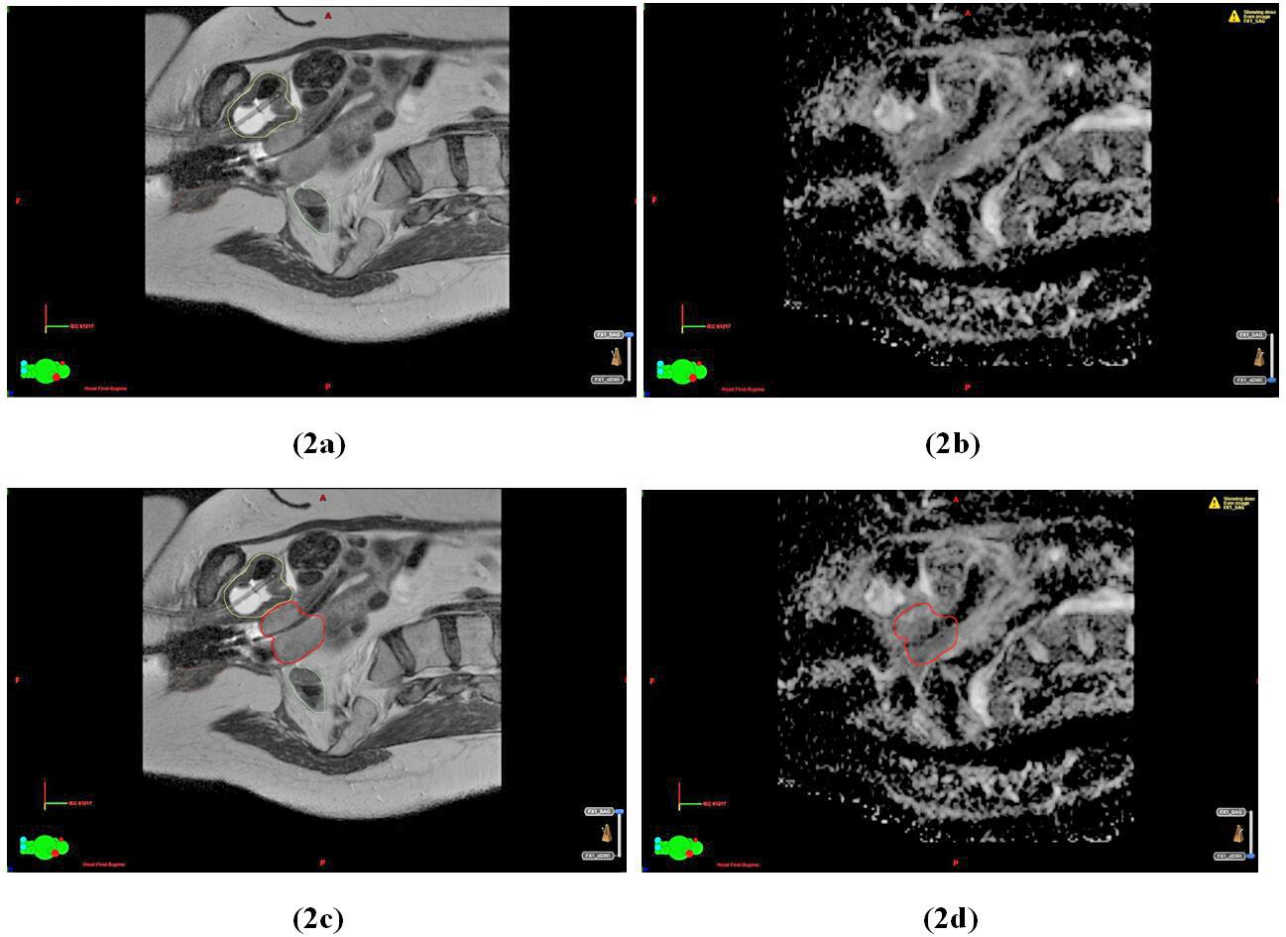
Another parasagittal slice in the T2W image dataset (a) and the corresponding slice in the ADC map (b) containing the tandem for Patient 1. The same slices in the T2W image dataset (c) and the ADC map (d) displayed with the contours for the GTV (in red). Organ‐at‐risk contours for bladder (yellow) and sigmoid (light green) are displayed on the T2W images (a) and (c).

Figure 1 demonstrates the use of the registered T2W images and ADC maps for target delineation for Patient 1, diagnosed with Stage IIB cervix cancer. Note in Fig. 1 the good agreement in target delineation between corresponding slices on the T2W image and ADC map. Figure 2 shows a different slice containing the tandem for the same image datasets, and demonstrates a potential pitfall of the ADC maps which is the presence of distortions due to the metal applicator. The borders of the target appear distorted and delineation on the ADC map was done with caution, especially for target slices near the titanium tandem. These distortions are local to regions immediately surrounding the tandem applicator, as demonstrated by the undistorted boundaries of the vertebral bodies and bladder in the ADC map. Note, due to the otherwise poor image quality of the ADC maps, the ADC maps cannot be used for the delineation of OARs, which is limited to the T2W images.

**Figure 3:**
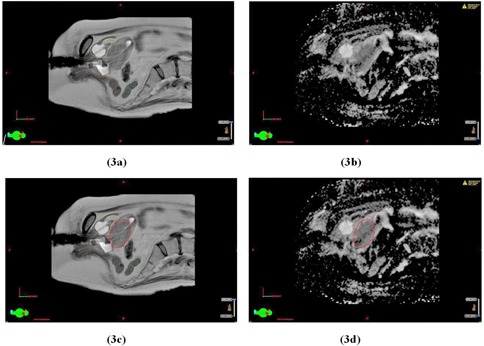
A parasagittal slice in the T2W image dataset (a) and the corresponding slice in the ADC map (b) for Patient 2. The same slices in the T2W image dataset (c) and ADC map (d) displayed with the contour for GTV (in red). Organ‐at‐risk contours for bladder (yellow), rectum (brown), and sigmoid (light green) are displayed on the T2W images (a) and (c).

Figure 3 shows the advantage of ADC maps for visualization of tumor borders for Patient 2, diagnosed with Stage IIB cervix cancer. In this case, the tumor borders on the T2W‐MRI were not very distinct, but become more obvious after verification against the ADC map.

### B. Examples of applicator reconstruction

**Figure 4:**
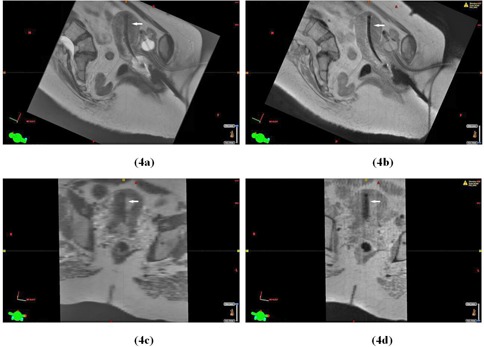
A parasagittal slice displaying the tandem (as indicated by the arrow) relative to the surrounding anatomy in the T2W‐MRI (a) and in the corresponding slice in the PDW‐MRI (b) for Patient 3. For the same dataset, a paracoronal slice displaying the tandem (as indicated by the arrow) in the T2W‐MRI (c) and in the corresponding slice in the PDW‐MRI (d).

Figure 4 displays parasagittal and paracoronal views of an implant for Patient 3 diagnosed with Stage IIIB cervix cancer. These views demonstrate how use of the PDW, registered to the T2W‐MRI, yields improved visualization of the tandem (dark) from the background (bright). In the reconstructed paracoronal views (Figs. 4(c) and 4(d)), the tandem is especially difficult to discern from the background in the T2W dataset due to the greater slice thickness (0.5 cm) needed to maintain acceptable signal in the T2W dataset. The higher signal achieved with PDW sequences allows the use of thinner slices (0.25 cm) and improved visualization in the reconstructed views. Note, however, due to the more limited soft tissue contrast, the PDW should be limited to applicator definition only.

### C. Examples of dose adaptation

**Figure 5:**
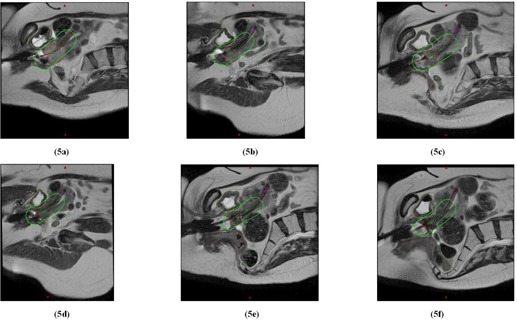
Parasagittal slices from T2W datasets for fractions 1–6 ((a)‐(f)) for Patient 1, displaying the prescription isodoses (in green) relative to the shrinking tumor volume (in red) and OARs (bladder in yellow, rectum in brown, and sigmoid in light green). Ovoids (not shown) were implanted and activated for all fractions. Weighting optimization was used for fractions 5–6.

Figure 5 displays an example of weighting optimization for Patient 1. For the first 4 fractions, the D2cc bladder was steadily increasing (i.e., from 100% to 119% of mean Point A dose) as the tumor responded to treatment, shifting the bladder closer to the applicator. The D2cc rectum varied between 80%–90% of mean Point A dose. By fraction 5, the GTV had decreased from an initial volume of 39.6 to 8.7 cc. The tumor shrinkage was considered to be adequate, and the bladder and rectal doses considered high enough to warrant de‐escalation of the dwell weights by 20% for fraction 5. The same magnitude of de‐escalation was applied for fraction 6 with the V100 at about 89%. These decreases in dwell weighting did help keep the cumulative D2cc for rectum and bladder at approximately $70\;{\text{Gy}}_{\alpha \beta 3}$ and $116\;{\text{Gy}}_{\alpha \beta 3}$, respectively. However, no further attempts were made to decrease the bladder D2cc in an effort to preserve target dose coverage.

**Figure 6:**
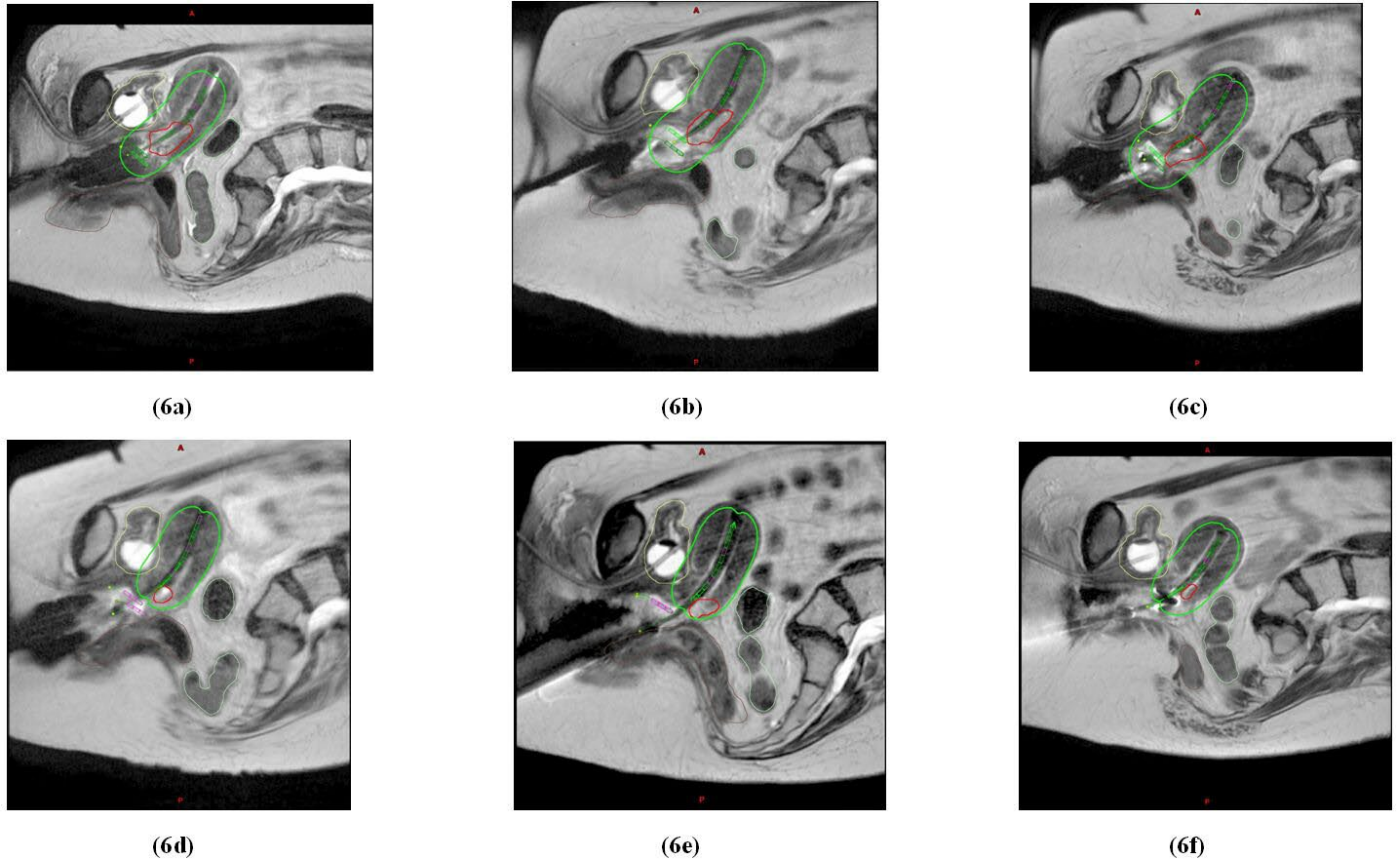
Parasagittal slices from T2W datasets for fractions 1–6 ((a)‐(f)) for Patient 3, displaying the prescription isodose (in green) relative to the shrinking tumor volume (in red) and OARs (bladder in yellow, rectum in brown, and sigmoid in light green). Ovoids were implanted for first 5 fractions, but not activated for fractions 4–5. No ovoids were implanted for the last fraction.

Figure 6 displays an example of applicator optimization for Patient 3. Because of anatomical changes that are typically observed as treatment progresses (e.g., tumor shrinkage and narrowing of vaginal fornices), the ovoid separation for Patient 3 decreased from 4.0 to 3.2 cm during the first 3 fractions, and consequently, the D2cc rectum increased from 80% to 106% of mean Point A dose. By fractions 4 and 5, the patient's anatomy pushed the ovoids to be too anterior, and as a result, the ovoids were not contributing significantly to GTV coverage, and were giving unnecessary dose to the OARs. Thus, the ovoids were not activated for fraction 4–5, decreasing rectal dose to about 40% of mean Point A dose. By fraction 6, the tandem was implanted only and was sufficient for GTV coverage. The modifications in applicator geometry did help keep the cumulative D2cc for rectum and bladder to be approximately $67\;{\text{Gy}}_{\alpha \beta 3}$ and $105\;{\text{Gy}}_{\alpha \beta 3}$, respectively, with the V100 at 100% for every fraction. Although not attempted for this patient, weighting optimization may have also been used for the last few fractions of this patient to attempt to reduce bladder D2cc even further because of the generous target coverage.

## IV. DISCUSSION

Our standard approach for cervix cancer treatment, PET/CT‐guided IMRT combined with either film‐based or CT‐based HDR ICBT, using standard source distribution rules normalized to Point A, has been performed for years.^(10)^ We have refined the approach for ICBT by incorporating advanced MRI techniques and automated dose tracking tools to perform dose adaptation during the brachytherapy treatment. Below, we discuss certain aspects of our approach within the context of GEC‐ESTRO recommendations and the experience of institutions that have adopted their recommendations.

With regard to MR imaging, GEC‐ESTRO describes the use of T2W‐MRI as the gold standard for tumor and critical structure visualization and as having sufficient contrast for applicator definition based on their experience with 0.2‐T and 1.5‐T MRI units, with the use of additional MRI sequences to be considered optional.^(23)^ While we also advocate the use of T2W‐MRI for ICBT, we recommend the use of additional MRI sequences for improved visualization of the target and critical structures using a combination of T2W imaging and DW‐ADC mapping and, in addition, for improved visualization of the applicator using PDW images.^11^, ^12^ Although we make no formal recommendations regarding the choice of MRI scanner for ICBT treatment planning, our brachytherapy technique is based on a 1.5‐T MRI scanner and may not readily apply to higher magnetic field strengths (i.e., 3‐T) due to the increased image distortion and susceptibility artifacts, in particular, those associated with a titanium applicator.^(24)^ A number of authors have described how titanium applicators, while MRI‐compatible, can also introduce susceptibility artifacts in the images, and that these artifacts are dependent on image sequence.^22^, ^24^ Therefore, similar to GEC‐ESTRO, we recommend that, in addition to CT scans, phantom MRI scans be acquired with clinical sequences as part of applicator commissioning. These scans should then be fused to one another to understand the nature of these artifacts and their effect on applicator geometry in the MR images.^(25)^

With regard to dose adaptation, Kirisits et al.^(8)^ describe a form of dose adaptation where one starts with standard source distribution rules normalized to Point A, and then performs symmetric, asymmetric, or more customized modifications of the dwell weights and positions with a ring and tandem applicator. Our dose adaptation approach is similar, but is currently limited to symmetric decreases of the dwell weights only to reduce the doses to OARs. Similar to our approach, Kirisits and colleagues used optimization to reduce OAR dose. However, a key difference from our approach is that the Kirisits study also optimized target dose coverage. The rationale behind our current dose adaptation scheme is to use the standard source loading patterns to treat the tumor, and if the GTV is reduced in volume by about 50%, to dose de‐escalate for the remaining 2 to 3 fractions to reduce OAR dose while maintaining at least 90% coverage of the GTV by prescription. Currently, the triggers for optimization in our technique are largely based on shrinkage of the GTV and also on OAR dose. Future development of our dose adaptation approach will include target dose optimization, and perhaps dose escalation, for those tumor volumes that do not respond over the course of treatment. This may involve varying individual dwell weights and dwell positions, similar to what was done by Kirisits et al.^(8)^ Potter et al.,^(9)^ who did implement the methods of the Kirisits study to perform MRI‐assisted dose‐volume adaptation, as well as dose escalation, reported in their single center an increase in local control and improvement in overall survival. Thus, there may be some benefit to incorporating target dose adaptation and dose escalation into our approach, as well.

GEC‐ESTRO recommendations for ICBT planning consider the dose‐volume coverage of a GTV as well as of a high‐risk clinical target volume (HR‐CTV), whereas our technique only tracks the GTV, defined as gross tumor and not including any expansions or “gray” zones.^(1)^ We chose to define GTV alone as the target volume as a simple, reproducible method for target definition using the DW–ADC maps in conjunction with T2W‐MRI. The utility of DW–ADC maps for identifying cancerous regions in the cervix has been demonstrated in the literature.^18^, ^19^, ^20^, ^21^ Preliminary work has shown good agreement (Dice Similarity Coefficient = 0.76 ± 0.06) between tumor volumes delineated on DW–ADC maps and on FDG‐PET images of cervix cancer patients.^(13)^ Dyk et al.^(14)^ showed that dose to the GTV from our treatment approach of combined MRI‐based HDR and PET/CT‐guided IMRT is highly correlated with local tumor control. GTV doses correlated with ≥90% local control included the D100 (>69 Gy), D90 (>98 Gy), and Dmean (>260 Gy). Thus, to ensure these levels of local control with our dose adaptation approach, we will continue to define the GTV, as described here, as the sole target of interest, and will also consider these GTV dose metrics when performing dose adaptation.

## V. CONCLUSIONS

Using our refined approach for MR‐based ICBT, we can perform multisequence imaging, automated dose tracking, and dose adaptation, and keep the time between implant and treatment delivery to within 2 hours. Although it may not be feasible for some clinics to implement all of the techniques as described here due to limited resources or established practices, we do believe that implementation of certain aspects of our technique will be of value to clinics performing MR‐based ICBT. Implementation of the multisequence MRI technique as described here will be of value for improved visualization of the target volume, critical structures, and applicator. Also, implementation of the dose tracking tools and dose adaptation technique by simply de‐escalating Point A‐based brachytherapy dose distributions will help balance target volume coverage with OAR sparing, without the need for more complex adaptation schemes.
